# Correction: A Novel Antifungal Is Active against *Candida albicans* Biofilms and Inhibits Mutagenic Acetaldehyde Production In Vitro

**DOI:** 10.1371/journal.pone.0101859

**Published:** 2014-07-03

**Authors:** The PLOS ONE Staff

## Notice of Republication

This article was republished on June 9, 2014, to correct the ordering of the images of Figures 1-3. The publisher apologizes for the error. Please download this article again to view the correct version. The originally published, uncorrected article and the republished, corrected article are provided here for reference.

## Supporting Information

File S1 Originally published, uncorrected article.
(PDF)

File S2 Republished, corrected article.
(PDF)
