# Supplementary material for: A Novel Antifungal Is Active against Candida albicans Biofilms and Inhibits Mutagenic Acetaldehyde Production In Vitro
Source: PLoS One. 2014 May 27;9(5):e97864. doi: 10.1371/journal.pone.0097864 (PMC4035295; doi:10.1371/journal.pone.0097864)
Supplement: Table S1 — List of primers used in this study. (DOCX) [file pone.0097864.s001.docx]

| **Table S1. List of primers used in this study.** | |
| --- | --- |
| **Primer** | **Sequence** |
| ADH1-F | TGTCCAAGCCGCTAAAATTC |
| ADH1-R | TATTGAACGGCCAAAGAACC |
| ADH2-F | AAATGGTTGAACGGCTCTTG |
| ADH2-R | GACGGTGACACCAGCACATAAG |
| ALD4-F | TTATGCCGTTGAATGTGCTC |
| ALD4-R | CTTTGCCCGTGATTTTATCAGC |
| ALD5-F | TGTTGTTACCGGTGGTGCTA |
| ALD5-R | CAACGGCTTCGTCAACAGTA |
| ALD6-F | TTACGTGGGTTACAAGTTGCTG |
| ALD6-R | GAGCTGCCATGATTATTTGTGA |
| ACS1-F | ATTTGCCAGCTTGGTTCATC |
| ACS1-R | CACCCTTTTTAACCCCCAAT |
| ACS2-F | CTCAAGGATTTTTCGGTCCA |
| ACS2-R | ATTCACCACCCAAAAACCAA |
| RIP1-F | TGTCACGGTTCCCATTATGATATTT |
| RIP1-R | TGGAATTTCCAAGTTCAATGGA |
