# Supplementary material for: Correction: A Novel Antifungal Is Active against Candida albicans Biofilms and Inhibits Mutagenic Acetaldehyde Production In Vitro
Source: PLoS One. 2014 Jul 3;9(7):e101859. doi: 10.1371/journal.pone.0101859 (PMC4081779; doi:10.1371/journal.pone.0101859)
Supplement: File S1 — Originally published, uncorrected article. (PDF) [file pone.0101859.s001.pdf]

A Novel Antifungal Is Active against *Candida albicans* Biofilms and Inhibits Mutagenic Acetaldehyde Production In Vitro

Mikko T. Nieminen[1,2,3,5], Lily Novak-Frazer[5], Vilma Rautemaa[5], Ranjith Rajendran[6], Timo Sorsa[2,4], Gordon Ramage[6], Paul Bowyer[5], Riina Rautemaa[1,3,4,5]*

1 Research Unit on Acetaldehyde and Cancer, University of Helsinki, Helsinki, Finland, **2** Department of Periodontology, Institute of Dentistry, University of Helsinki, Helsinki, Finland, **3** Department of Bacteriology and Immunology, Haartman Institute, University of Helsinki, Helsinki, Finland, **4** Department of Oral and Maxillofacial Diseases, Helsinki University Central Hospital Finland, Helsinki, Finland, **5** The University of Manchester, Institute of Inflammation and Repair, Manchester Academic Health Science Centre, University Hospital of South Manchester, Wythenshawe Hospital, Manchester, United Kingdom, **6** Infection and Immunity Research Group, Glasgow Dental School and Hospital, School of Medicine, College of Medicine, Veterinary and Life Sciences, University of Glasgow, Glasgow, United Kingdom

Abstract

The ability of *C. albicans* to form biofilms is a major virulence factor and a challenge for management. This is evident in biofilm-associated chronic oral-oesophageal candidosis, which has been shown to be potentially carcinogenic *in vivo*. We have previously shown that most *Candida* spp. can produce significant levels of mutagenic acetaldehyde (ACH). ACH is also an important mediator of candidal biofilm formation. We have also reported that D,L-2-hydroxyisocaproic acid (HICA) significantly inhibits planktonic growth of *C. albicans*. The aim of the present study was to investigate the effect of HICA on *C. albicans* biofilm formation and ACH production *in vitro*. Inhibition of biofilm formation by HICA, analogous control compounds or caspofungin was measured using XTT to measure biofilm metabolic activity and PicoGreen as a marker of biomass. Biofilms were visualised by scanning electron microscopy (SEM). ACH levels were measured by gas chromatography. Transcriptional changes in the genes involved in ACH metabolism were measured using RT-qPCR. The mean metabolic activity and biomass of all pre-grown (4, 24, 48 h) biofilms were significantly reduced after exposure to HICA ($p<0.05$) with the largest reductions seen at acidic pH. Caspofungin was mainly active against biofilms pre-grown for 4 h at neutral pH. Mutagenic levels (>40 μM) of ACH were detected in 24 and 48 h biofilms at both pHs. Interestingly, no ACH production was detected from D-glucose in the presence of HICA at acidic pH ($p<0.05$). Expression of genes responsible for ACH catabolism was up-regulated by HICA but down-regulated by caspofungin. SEM showed aberrant hyphae and collapsed hyphal structures during incubation with HICA at acidic pH. We conclude that HICA has potential as an antifungal agent with ability to inhibit *C. albicans* cell growth and biofilm formation. HICA also significantly reduces the mutagenic potential of *C. albicans* biofilms, which may be important when treating bacterial-fungal biofilm infections.

Citation: Nieminen MT, Novak-Frazer L, Rautemaa V, Rajendran R, Sorsa T, et al. (2014) A Novel Antifungal Is Active against *Candida albicans* Biofilms and Inhibits Mutagenic Acetaldehyde Production In Vitro. PLoS ONE 9(5): e97864. doi:10.1371/journal.pone.0097864

Editor: Martine Bassilana, Université de Nice-CNRS, France

Received January 30, 2014; **Accepted** April 25, 2014; **Published** May 27, 2014

Copyright: © 2014 Nieminen et al. This is an open-access article distributed under the terms of the Creative Commons Attribution License, which permits unrestricted use, distribution, and reproduction in any medium, provided the original author and source are credited.

Funding: MN received funding from Yrjö Jahsson Foundation (www.yjs.fi), Orion Research Foundation (http://www.orion.fi/Tutkimus-ja-tuotekehitys/Orion-Farmos-Tutkimussaatio/) and Finnish Dental Society Apollonia (www.apollonia.fi). R. Rautemaa, VR and LNF were supported by the National Aspergillosis Centre, UK (www.uhsm.nhs.uk/nac/Pages/default.aspx). PB received funding from EU framework 7 (http://cordis.europa.eu/fp7/home_en.html). TS was supported by Helsinki University Central Hospital Research Foundation (http://www.hus.fi/tutkijalle/tutkimusrahoitus/Sivut/default.aspx). GR and R. Rajendran received no funding towards this piece of work. The funders had no role in study design, data collection and analysis, decision to publish, or preparation of the manuscript.

Competing Interests: I have read the journal's policy and have the following conflicts: TS is one of the inventors of European Patent Office patent EP0871438B1: "Use of alpha-hydroxy acids in the manufacture of a medicament for the treatment of inflammation". The patent is owned by Extracta Oy Ltd., Helsinki, Finland. TS has not received any royalties regarding this patent. GR has acted as a consultant and a speaker for Gilead Sciences, Astellas, Merck Sharp & Dohme, GlaxoSmithKline, BluTest Laboratories and Fulhold Ltd. This does not alter our adherence to all the PLOS One policies on sharing data and materials.

* E-mail: riina.richardson@manchester.ac.uk

Introduction

C. albicans is the most common fungal pathogen in humans causing both superficial and systemic infections [1]. The ability of *C. albicans* to form biofilms is a major virulence factor and approximately 65% of microbial infections in humans are biofilm-related [2,3]. This mode of growth protects *Candida* spp. against endogenous and exogenous inhibitory substances. High antifungal tolerance is a well-known factor of *Candida* biofilms and a challenge for treatment [4]. The surrounding environment and its cues have a major impact on *C. albicans* biofilm formation. Hyphal growth is an essential element of *C. albicans* biofilms that provides integrity within these complex and dense structures [5,6]. Multiple studies have shown that environmental pH, oxygen levels and nutritional status impact on the morphogenesis of *C. albicans* [7–10].

Chronic biofilm infections cause inflammation, which is linked to carcinogenesis [11]. Biofilm related chronic mucocutaneous candidosis (CMC) has been associated with a significant risk for oral cancer *in vivo* [12–14]. *C. albicans* isolated from these patients are able to produce high levels of carcinogenic acetaldehyde (ACH) *in vitro* thus providing one possible explanation for carcinogenesis [15]. The International Agency for Research on Cancer (IARC) has classified ACH associated with alcoholic beverages as a GROUP I carcinogen [16]. Our group has

previously shown that planktonically grown *Candida* spp. can produce significant and mutagenic levels of ACH (>40 μM) as part of their ethanol metabolism *in vitro* [17,18]. ACH is an intermediate product of the pyruvate bypass pathway that converts pyruvate into acetyl-CoA in the cytosol under low oxygen tension or anaerobic conditions during fermentation (Fig. 1A; [19]). In bypass, pyruvate is first converted to ACH by pyruvate decarboxylase (Pdc) and then ACH is metabolized either to acetate by aldehyde dehydrogenases (Ald) or turned into ethanol by alcohol dehydrogenase (Adh) enzymes. Acetate is further metabolized to acetyl-CoA by acetyl CoA synthetase (Acs) enzymes and transported into mitochondria by a carnitine dependent mechanism.

Genomic databases show that there are five putative genes encoding Adh enzymes for *C. albicans* [20]. *ADH1* encodes a cytosolic Adh1p enzyme that is the only isoenzyme studied extensively and the protein is able to function bi-directionally in ACH-ethanol conversion thus making the use of ethanol as a carbon and energy source possible [21]. Proteomic and transcriptional studies have shown that *ADH1* is one of the factors that controls biofilm formation and growth [22,23]. Recently more information has been revealed about other Adh isoenzymes. In *S. cerevisiae* Adh2p functions in glucose-depleted conditions, and converts ethanol to ACH *in vitro* [24]. In *C. albicans*, *ADH2* is upregulated in planktonic cells grown in hypoxic conditions and mainly in the stationary growth phase [8,25]. *ADH5* has a role in controlling the biofilm matrix formation in *C. albicans* [26].

The number and role of Ald enzymes in *C. albicans* are not well known, but in *S. cerevisiae* there are five known genes [27]. The mitochondrial isoforms in *S. cerevisiae* are encoded by *ALD4* (major) and *ALD5*. The cytosolic counterparts are encoded by *ALD2, ALD3* and *ALD6* (major). In *C. albicans*, the protein encoded by

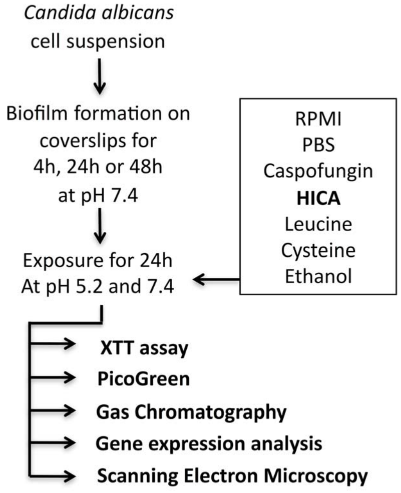

Figure 1. Relative expression of genes related to ACH metabolism. (A) A schematic model of central carbon metabolism incorporated with genes of interest within ethanol metabolism (abbreviations as follows: G6P = glucose-6-phosphate, KICA = α-ketoisocaproic acid). (B) Heat map panel of gene expression in *C. albicans* biofilms at three different stages of growth at pH 5.2 and 7.4 after 24 h exposure to PBS, caspofungin, HICA, leucine, cysteine or ethanol. Fold changes are expressed relative to control (RPMI) at the corresponding time point. Black represents no change in expression, green is up-regulation, and red is down-regulation. A brighter color indicates greater degree of change in expression. Relative gene expressions were calculated by the Pfaffl method using the REST 2009 software provided by Qiagen [63].
doi:10.1371/journal.pone.0097864.g001

ALD4 is known to function in carnitine biosynthesis in the cytosol [28]. The carnitine biosynthesis and carnitine-dependent transformation of Acetyl-CoA are vital for growth on non-fermentable carbon sources and contribute to biofilm formation [29]. *ALD5* is upregulated in *C. albicans* biofilms exposed to oxidative stress and hypoxia [30–32]. Our group has shown that *ALD6* together with *ACS1* control the accumulation of ACH in planktonic *C. albicans* cultures in hypoxia [25]. Acs enzymes have been studied extensively in *C. albicans*. *ACS2* is essential for growth and *ACS1* is necessary for utilization of alternative carbon sources [33]. Recent studies show that proteins linked to fermentation are abundant in hyphae and biofilms [30,34,35]. Upregulation of fermentation has been shown in *C. albicans* colonization *in vivo* [36]. Altogether, these facts underline the importance of pyruvate bypass genes and fermentation for biofilm formation.

Poor efficacy and patient compliance and various side effects of commonly used antifungals have been major problems in the management of *Candida* infections [1]. The most promising chemotherapeutic approaches against *Candida* biofilms are observed with the echinocandin class of antifungals, which are non-competitive inhibitors of (1,3)-β-D-glucan synthase, an essential enzyme in cell wall synthesis and integrity [37,38]. Caspofungin is the most extensively used echinocandin, especially in treatment for invasive candidosis [39,40]. Although caspofungin has proven effective against *C. albicans* biofilms, a paradoxical effect on growth by induction of chitin synthesis and decreased susceptibility have been noted, suggesting there are limitations in its use [41,42].

Recent studies from our group show promising efficacy of D,L-2-hydroxyisocaproic acid (HICA) against the growth of a spectrum of planktonically grown pathogenic bacteria and fungi including *C. albicans*, *C. glabrata* and *C. tropicalis* [43,44]. HICA is a α-hydroxy-aminoacid and a leucine metabolite, which is produced by *Lactobacillus* species and also found in human muscle and connective tissues [45–47]. It has been used as a nutritional supplement by professional athletes and industrially as animal feeds [47,48]. Therefore it is proven to have a good biocompatibility and safety profile. In addition, there is evidence that HICA has anti-inflammatory properties through inhibition of host extracellular matrix degrading proteases *in vitro* [49]. Multiple studies have shown that *Lactobacillus* spp. metabolites which are similar to HICA and other amino acid derivatives inhibit fungal growth [45,50–52].

In this study our aim was to determine the ability of HICA to inhibit biofilm formation and to reduce the mutagenic potential of *C. albicans* biofilms through changes in ACH metabolism *in vitro*. We also wanted to show visually the impact of HICA on biofilm ultrastructure and examine further the effect of caspofungin and HICA-related carbon sources on biofilm formation and ACH metabolism. Our hypothesis is that the formation of *C. albicans* biofilms is strongly reduced and damaged by HICA and furthermore the mutagenic potential is significantly decreased by the induction of genes involved in pyruvate bypass and ACH metabolism.

Materials and Methods

Study design

Biofilms were grown on coverslips in RPMI medium at 37°C at pH 7.4 for 4, 24 and 48 h (Fig. 2; [53,54]). Pre-grown biofilms were exposed to HICA, leucine, cysteine or ethanol for 24 h at pH 5.2 or pH 7.4. Leucine was included as a metabolic comparator. Cysteine is toxic to fungi in high concentrations similar to HICA and was included as a comparator. Ethanol is the end product of fermentation and main source of ACH.

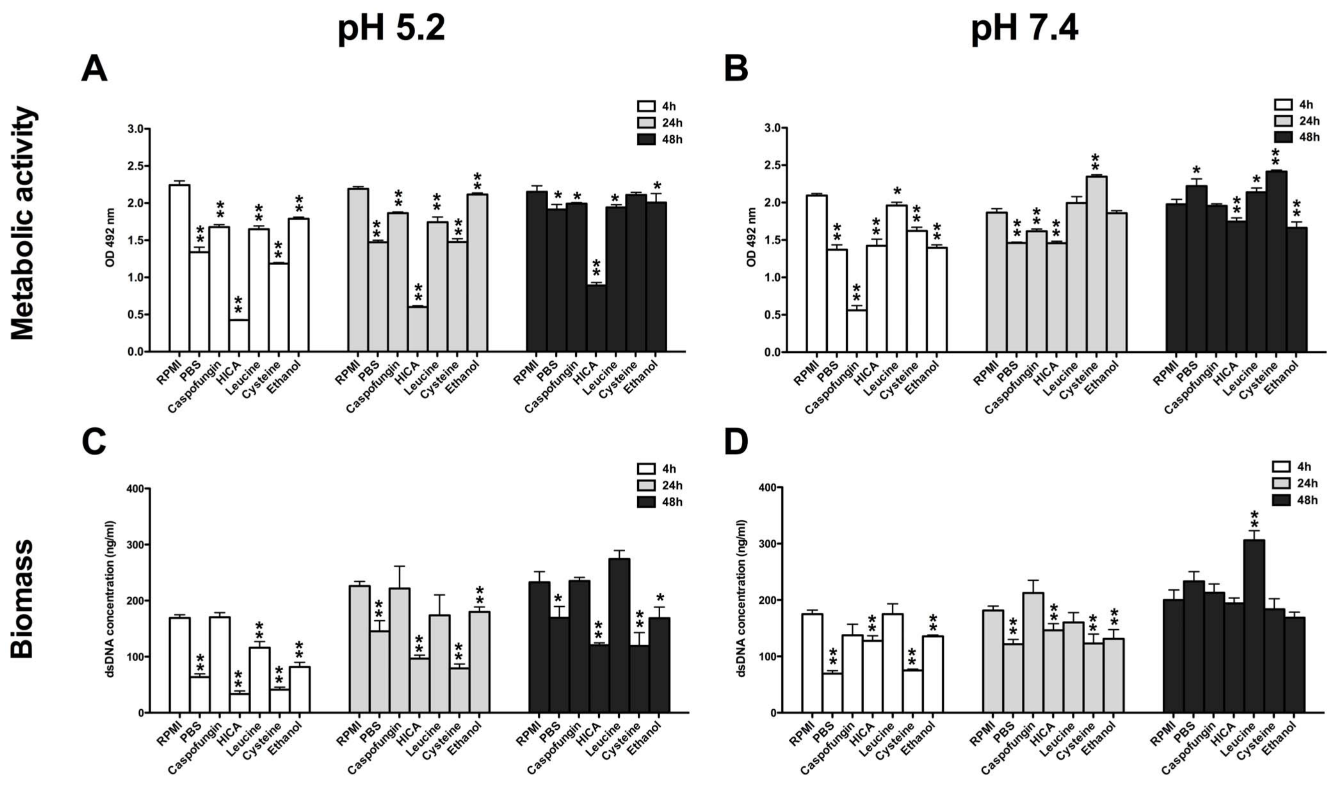

Figure 2. Summary of study design.
doi:10.1371/journal.pone.0097864.g002

Caspofungin was used as an antifungal comparator, and PBS and RPMI as controls. Five different methods were used to evaluate the effects of these substances on *C. albicans* biofilm formation and mutagenic properties.

XTT assay was used to determine cellular metabolism, and the amount of double stranded DNA (dsDNA) was used to reflect biofilm biomass [55–58]. ACH production in glucose or ethanol was measured by gas chromatography. To detect changes in ACH metabolism, gene expression of seven target genes (*ADH1, ADH2, ACS1, ACS2, ALD4, ALD5* and *ALD6*) and one reference gene (*RIP1*) was analyzed by quantitative real time PCR (RT-qPCR). Scanning electron microscopy (SEM) was used to visualize the impact of HICA on biofilm structure. All experiments were done twice in triplicate. In total 1,300 biofilms were used in the study.

Strain and culture conditions

C. albicans laboratory strain SC5314 was used [20,59]. The strain was stored at −80°C and plated twice on Sabouraud dextrose agar (Melford, UK) and incubated at 37°C for 48 h before use to check viability and purity. One colony was then inoculated into 20 ml yeast peptone dextrose (YPD) broth (Melford, UK) and grown overnight at 37°C with continuous shaking. The cells were harvested, washed twice with sterile phosphate-buffered saline (PBS) (Sigma-Aldrich, USA) and then re-suspended in RPMI-1640 (Sigma-Aldrich) supplemented with L-glutamine and buffered to pH 7.4 with morpholinepropanesulfonic acid (MOPS) (Oxoid Ltd., UK). The cell density was measured using a haemocytometer and adjusted to 1.0×10^{6} cells/ml to produce the standardized biofilm inoculum.

Biofilm growth and treatments

C. albicans biofilms were grown on Thermanox coverslips (Thermo Scientific Nunc, USA) in RPMI-1640 medium containing 2 g/l D-glucose (#6504; Sigma-Aldrich, USA) at pH 7.4 in static 24-well plates (Corning CoStar, USA) for 4, 24 or 48 h at 37°C. The biofilms were then exposed to 5% (w/v) HICA (TCI Europe, Belgium), 10 mg/l caspofungin (Merck & Co., USA), 5% (w/v) leucine (L-leucine Sigma-Aldrich, USA), 5% (w/v) cysteine (L-cysteine, Sigma-Aldrich, USA), 0.05% (v/v; 11 mM) ethanol, PBS or RPMI-1640 #6504 for 24 h at pH 5.2 or 7.4 at 37°C. Substrates were dissolved in RPMI-1640 #6504 except for the ethanol treatment, which was dissolved in RPMI-1640 #1383 without D-glucose or sodium bicarbonate and then adjusted to pH 5.2 or 7.4.

XTT-assay and dsDNA measurements

Before analysis, biofilms were washed with sterile PBS and then transferred into fresh 24-well plates. Then 200 µl saturated XTT/1 µM menadione solution was added to the biofilms, the plates covered with aluminium foil and transferred to a 37°C incubator for 2 h. After incubation, 100 µl of the XTT supernatant was transferred into fresh 96-well plates and the colorimetric changes were measured spectrophotometrically (BMG Labtech, UK) at 490 nm.

Fluorescent nucleic acid Quant-iT PicoGreen dsDNA reagent (Molecular Probes Inc., USA) was used for quantifying dsDNA in solution. Briefly, DNA was extracted from the biofilms using a modified cetyltrimethylammonium bromide (CTAB) method [60]. The DNA and PicoGreen reagent were mixed thoroughly in the well before fluorometric analysis at 492 nm (BMG Labtech, UK). The lambda DNA within the Quant-iT kit was used to construct the standard curve (concentration range 40–500 ng/ml) according

to the manufacturer's instructions and measured alongside the samples (100 µl per well; Corning Costar, UK).

Acetaldehyde measurements

ACH production was measured by headspace gas chromatography using a previously described method [18]. Briefly, gas chromatography was performed with a Varian CP-3800 equipped with a Zebron ZB-WaxPlus column (30 mx0.32 mmx0.5 µm, Phenomenex, UK), and CombiPal autosampler (CTC Analytics GmbH, Germany). Peaks were detected by flame ionization.

Biofilms were washed once with PBS post-exposure (24 h in 5% (w/v) HICA, 0.05% (v/v) ethanol, 10 mg/l caspofungin or RPMI) and transferred into 20 ml glass vials containing 1 ml 11 mM (0.05% (v/v)) ethanol, 100 mM (1.8% (w/v)) D-glucose or PBS (control). The samples were sealed with silicon caps and incubated at 37°C for 30 min. The reaction was stopped by the addition of 100 µl of perchloric acid (PCA, final concentration 0.6 M). To measure baseline or artefactual ACH production, 100 µl PCA was added immediately after transferring the biofilms into vials and prior to adding ethanol, D-glucose or PBS substrate.

Gene expression analysis

Exposed biofilms were flash-frozen in liquid nitrogen, then stored at −80°C until RNA extraction. Biofilms were detached by vortexing and then homogenized using glass beads (425–600 µm in diameter; Sigma, UK) and a FastPrep Instrument (MP Biomedical, USA). The samples were centrifuged at RT for 1 min at 12,000 *g* after the FastPrep step. RNA extractions were performed using the ISOLATE I RNA Mini kit (Bioline, UK) and genomic DNA was removed using Ambion DNA-free DNAse (Life Technologies, UK) treatment according to the manufacturer's instructions. RNA was quantified and quality assessed by spectroscopy (A_{260nm}) and the purity was further determined by RT-qPCR and gel electrophoresis. RNA was stored at −80°C for further analysis.

cDNA was synthesized using the AffinityScript QPCR cDNA synthesis kit (Agilent, UK) following the manufacturer's directions using 6 µl of RNA (10 ng/µl) as a template. In no-RT controls reverse transcriptase was replaced by water. Reverse transcription was carried out at 25°C for 5 min, then at 42°C for 60 min and finally at 95°C for 5 min. RT-qPCR reactions were prepared using the Brilliant II SYBR Green 2-step kit (Agilent, UK) with the reaction containing 6.25 µl SYBR Green master mix, 1 µl Forward (F) primer, 1 µl Reverse (R) primer, 2 µl cDNA and 2.25 µl DNA/RNA-free molecular grade water. PCR was performed with a Stratagene MX3005P instrument (Agilent, UK). For all RT-qPCR experiments, primers were designed using Primer 3.0 Plus [61] and are shown in Table S1. *RIP1* was used as a reference gene [62]. The results shown are an average of triplicates from two independent biological samples. The PCR amplification efficiencies of the primers for all target genes were optimized prior to analysis. The relative gene expression was calculated by the Pfaffl method using the REST 2009 software provided by Qiagen [63]. The gene expression results were compiled to a heatmap using Microsoft Excel 2010 ver. 14.0.

Scanning electron microscopy of *in vitro* biofilms

Biofilms were grown in RPMI as previously described for 24 h and then exposed to 5% (w/v) HICA or RPMI #6504 (control) for 24 h at pH 5.2 or pH 7.4. The biofilms were washed once with PBS before being placed in fixative (2% (v/v) paraformaldehyde, 2% (v/v) glutaraldehyde, 0.15 mM sodium cacodylate and 0.15% (w/v) Alcian blue in PBS) overnight as previously described [64]. Biofilms were then rinsed in 0.1 M phosphate buffer and air-dried in desiccators. Notably, harsh dehydration steps were not performed to minimize the damage to the original biofilm structure. The samples were coated with gold/palladium (40%/ 60%) and observed under a scanning electron microscope (Leo 435 VP) in high vacuum mode at 10 kV.

Statistical analysis

Statistical analyses for XTT, PicoGreen and ACH measurements were carried out using GraphPad Prism ver. 5.0 (GraphPad Software Inc., La Jolla, CA, USA) and SPSS ver. 18.0 (SPSS Inc., Chicago, IL, USA). A generalised estimating equations (GEE) model was used for comparisons of XTT, PicoGreen and ACH results. Statistical comparisons for gene expression analyses were performed using a pair-wise fixed reallocation test within REST 2009 software (Qiagen, CA, USA). $P<0.05$ was considered statistically significant. Basal transcription of genes in each condition was calculated relative to the reference gene, *RIP1*, using equation $2^{-\Delta Ct}$ ($\Delta Ct = Ct_{Treatment}$-$Ct_{Reference}$). A 2-tailed Spearman's rho (r_s) with a 95% confidence interval was used for the analyses of correlations. Basal expression levels were used for the analyses of correlations. The results are expressed as means (±SEM).

Results

HICA has a significant inhibitory effect on biofilm formation at acidic pH

At acidic pH, the metabolic activity (MA) of all pre-grown biofilms was significantly reduced by HICA exposure (Fig. 3A; 4, 24 and 48 h, $p<0.001$): MA was reduced by 59–81% compared to RPMI at pH 5.2. A maximum drop of 81% in MA was measured in 4 h biofilms post-exposure to HICA (OD_{492}; 0.43±0.01 vs. 2.24±0.06 in RPMI). MA was reduced in all pre-formed biofilms under all treatment conditions except for exposure to cysteine, which had no effect on the MA of 48 h biofilms. Similar levels of MA were measured for 4, 24, and 48 h biofilms in the control condition, RPMI (Fig. 3A, B).

At neutral pH, MA was reduced in all pre-formed biofilms compared to RPMI, except after exposure to cysteine and leucine where an increase was observed in 24 and 48 h biofilms (Fig. 3B). Caspofungin exposure had the greatest negative impact on MA of all conditions in 4 h biofilms (OD_{492} 0.56±0.06 vs. 2.09±0.03 in RPMI, $p<0.001$), but a much lower reduction was seen in 24 and 48 h biofilms. MA was lower by 12–32% and the reduction was highly significant in all pre-formed biofilms after exposure to HICA ($p<0.001$).

Biofilm biomass was significantly decreased after all treatments in all biofilms pre-grown for 4, 24 and 48 h at pH 5.2, except after exposure to caspofungin (Fig. 3C; $0.001<p<0.05$). Leucine exposure increased the biomass of 48 h biofilms, but this change was not statistically significant (p = ns). Biomass was greatly affected by HICA and cysteine exposure at acidic pH (overall reduction was by 62% and 63%, respectively). The greatest reduction in biomass (80%) was observed in 4 h biofilms after exposure to HICA (dsDNA conc. 33.4±5.6 ng/µl vs. 169.1±5.7 ng/µl in RPMI). Caspofungin had no significant effect on biomass at pH 5.2, with the highest reduction being only 2% measured in 24 h pre-formed biofilms (221.5±39.8 ng/µl vs. 226.1±8.2 ng/µl in RPMI). Biomass of pre-grown biofilms was reduced by 42% post-exposure to PBS.

Upon exposure to PBS, HICA, cysteine or ethanol at neutral pH, biofilm biomass was reduced significantly in 4 and 24 h pre-formed biofilms (Fig. 3D, $0.001<p<0.05$). Exposure to caspofungin or leucine had no discernable effect on biomass of any pre-

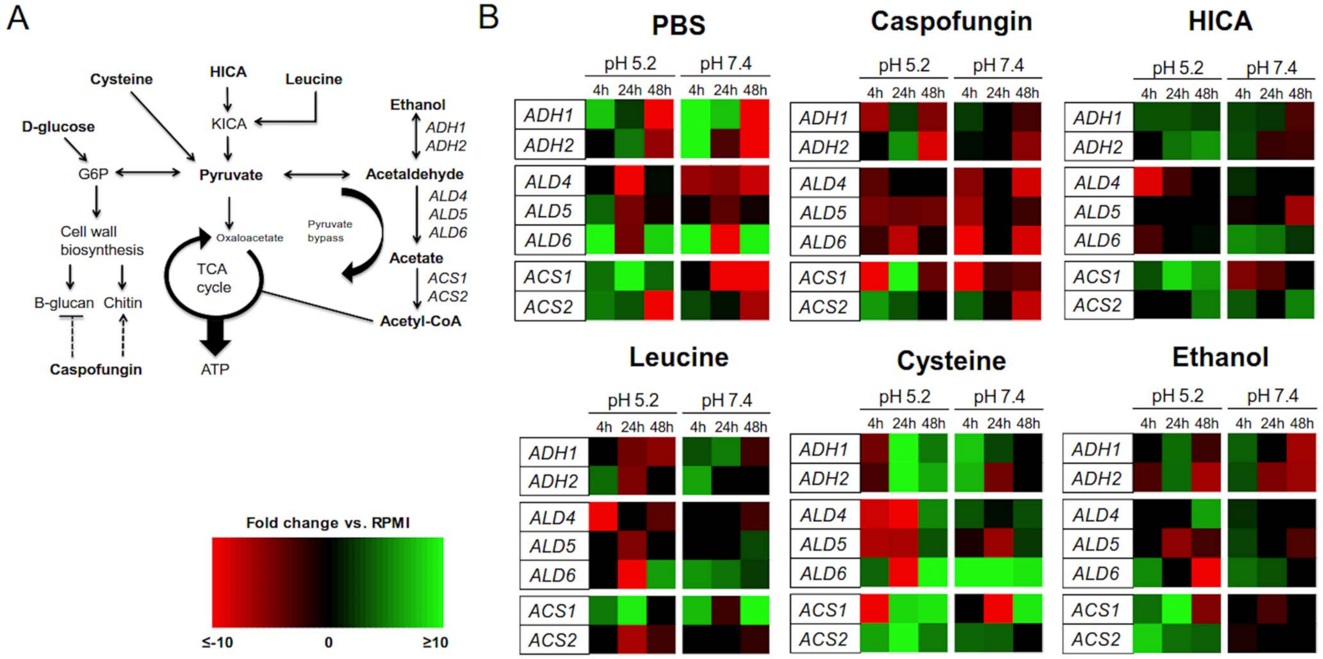

Figure 3. Changes in metabolic activity and biomass of *C. albicans* biofilms. Metabolic activities measured by XTT-assay (panel A, B) and dsDNA levels (panel C, D) reflecting the biomass of *C. albicans* biofilms at pH 5.2 and 7.4. Biofilms were grown for 4, 24 or 48 h in RPMI at pH 7.4 and then exposed to PBS, caspofungin, HICA, leucine, cysteine or ethanol for another 24 h. Values were measured twice in triplicate and expressed as mean (±SEM). Means were compared to the control treatment (RPMI). Statistically significant differences were calculated using a GEE-model and were marked (**p<0.001,* 0.001<p<0.05).
doi:10.1371/journal.pone.0097864.g003

grown biofilms. The biomass of 4 h and 24 h pre-formed biofilms was reduced by 27% and 19% respectively after exposure to HICA when compared to RPMI (Fig. 3D; 127.6±8.9 ng/µl and 146.2±11.9 ng/µl vs. 174.9±7.1 ng/µl and 181.5±7.7 ng/µl respectively). Leucine exposure had a significant effect only on 48 h biofilm biomass, which was increased by 53% (p<0.001; 306.1±17.0 ng/µl vs. 200.1±17.7 ng/µl respectively).

Acetaldehyde catabolism was induced in biofilms exposed to HICA at acidic pH

High basal expression (relative to the reference gene, *RIP1*) of *ADH1*, *ADH2* and *ALD5* in pre-formed biofilms exposed to pH 5.2 suggested that expression of these genes was indicative of significant biological function in the biofilm. *ALD4, ALD6, ACS1 and ACS2* were expressed at very low basal level, therefore changes in expressions of these genes should be treated with caution. *ALD5* was highly expressed in all pre-formed biofilms after all exposures including the RPMI control. *ADH1* was also highly expressed with respect to the reference gene in 4 and 48 h biofilms, but not to the same extent in 24 h biofilms. High *ADH1* expression was correlated with high *ADH2* expression in the following conditions: 4 h biofilms exposed to cysteine, leucine or PBS and 48 h biofilms exposed to cysteine or HICA.

When relative gene expression was compared, between treatment and control conditions, *ADH1* was found to be up-regulated 1.8–2.1 fold after HICA exposure and down-regulated by 0.2–0.3 fold after caspofungin exposure in 4 and 48 h pre-formed biofilms (Fig. 1B). In addition, *ALD5* was significantly down-regulated in all pre-formed biofilms after caspofungin and ethanol exposure (0.3–0.8 fold), but not affected by exposure to HICA. Generally, *ACS1* was down-regulated (0.02 to 0.4 fold) in 4 and 48 h pre-formed biofilms after caspofungin exposure but up-regulated (2 to 4 fold) after HICA exposure. Cysteine exposure resulted in induced expression of all genes in 48 h pre-formed biofilms.

Acetaldehyde catabolism was also induced in biofilms exposed to HICA at neutral pH

The high basal expression of *ALD5* observed in biofilms at pH 5.2 was also observed in all pre-formed biofilms grown at pH 7.4, for all exposures including RPMI control biofilms. Overall a decrease in transcription rates in biofilms at later stages of development was observed at neutral pH compared to transcription rates at acidic pH. Unlike basal transcription at pH 5.2, *ADH1* and *ADH2* were generally poorly expressed at pH 7.4.

When biofilm gene expression was compared relative to the biofilm controls (RPMI), more up-regulation was observed in biofilms exposed to HICA than in biofilms exposed to caspofungin (pH 7.4, Fig. 1B). In general, heatmaps for biofilms exposed to leucine, ethanol and cysteine showed a similar pattern to that of HICA, except for expression of *ALD5* and *ACS1*. Cysteine and leucine exposure led to higher expression of *ALD5* and *ACS1* in later biofilms compared to HICA and caspofungin.

Mutagenic potential of *C. albicans* biofilms is reduced by HICA at acidic and neutral pH

Generally, the highest ACH production was observed in control biofilms (RPMI); this was evident after incubation in 0.05% ethanol (Fig. 4C, D; 135.2±5.7 µM at 24 h, pH 5.2 and 130.3±12.3 µM at 48 h, pH 7.4). In D-glucose, ACH levels for biofilms exposed to RPMI were markedly lower but still above the mutagenic level of 40 µM at neutral pH (Fig. 4B; 53.8±1.9 µM at 48 h). No ACH was detected in any condition or pH when biofilms were incubated with PBS.

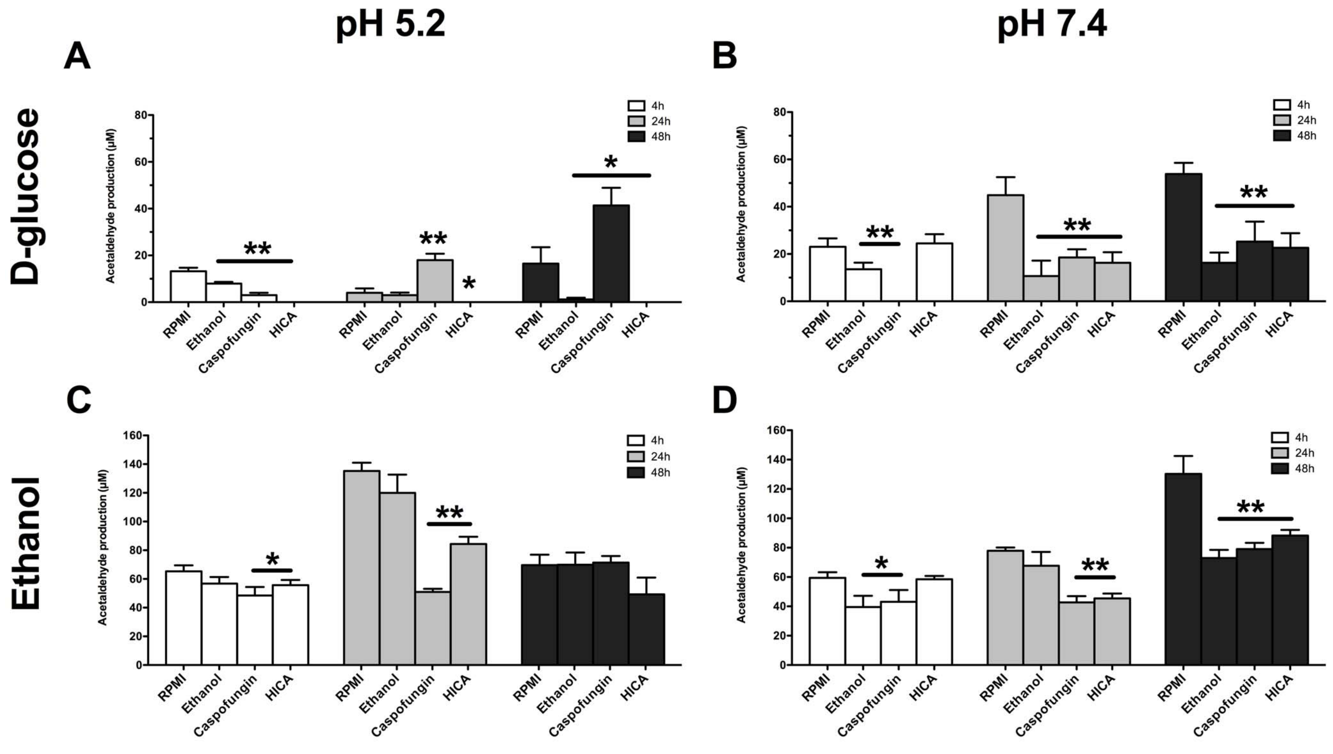

Figure 4. ACH production by *C. albicans* biofilms. Mean (±SEM) ACH production by *C. albicans* biofilms. Biofilms at three different stages of growth (4 h, 24 h and 48 h), were incubated for 30 min at 37°C with 100 mM D-glucose (A,B) or 0.05% ethanol (C,D) after 24 h exposure to RPMI, ethanol, caspofungin or HICA at pH 5.2 or 7.4. Values were measured twice in triplicate and means were compared at each time point to the control treatment (RPMI). Statistically significant differences were calculated using a GEE-model and were marked (**$p<0.001$, * $0.001<p<0.05$).
doi:10.1371/journal.pone.0097864.g004

ACH was not detected in biofilms exposed to HICA at acidic pH after incubation with D-glucose (Fig. 4A). However, under the same conditions, ACH levels increased significantly compared to RPMI in 24 h and 48 h pre-grown biofilms after caspofungin exposure ($p=0.021$ and $p=0.01$, respectively), with the highest ACH production detected in 48 h biofilms (Fig. 4A; 41.3±7.5 µM). Generally, low production of ACH from D-glucose was observed in pre-grown biofilms exposed to ethanol at both pHs, compared to control biofilms (Fig. 4A, B).

At neutral pH no ACH was produced during incubation with D-glucose by biofilms pre-grown for 4 h and exposed to caspofungin compared to all other exposures, including the control (RPMI). However, ACH production among 24 h and 48 h pre-formed biofilms exposed to caspofungin, HICA or ethanol was similar but significantly lower than in control biofilms ($p<0.001$, Fig. 4B). Equivalent levels of ACH were produced from ethanol by biofilms exposed to RPMI and ethanol at acidic pH (Fig. 4C). HICA or caspofungin exposure reduced the ACH production from ethanol in 4 and 24 h but not 48 h pre-formed biofilms at pH 5.2 (Fig. 4C, $p<0.05$). At neutral pH, HICA or caspofungin exposure led to significantly lower ACH production from ethanol in 24 and 48 h pre-formed biofilms (Fig. 4D; $p<0.001$). Although caspofungin exposure also decreased ACH production in 4 h pre-formed biofilms under these conditions, this was not the case after HICA exposure. In ethanol incubation ACH levels were generally above the mutagenic level (40 µM) at both pHs, although reductions close to this level were seen with HICA and caspofungin.

Gene expression reflected the changes in ACH levels

Correlations were calculated for genes that exhibited a high basal level of expression (*ADH1*, *ADH2*, *ALD5*). *ALD5* expression correlated with pH in all pre-formed biofilms exposed to HICA ($r_s=0.7257$, $p<0.001$), caspofungin ($r_s=0.695$, $p=0.0014$) and PBS ($r_s=0.501$, $p=0.034$). In addition, in biofilms exposed to PBS, expression of both *ADH1* and *ADH2* correlated significantly with pH, similar to *ALD5* ($r_s=0.685$, $p=0.0017$ for *ADH1* and $r_s=0.769$, $p<0.001$ for *ADH2*, respectively). This implies that downstream ACH metabolism is controlled similarly at both pHs. No correlations were observed between gene expression and pH for other exposure conditions. *ADH1* expression correlated positively with *ADH2* expression ($r_s=0.703$, $p<0.001$).

Changes in gene expression mirrored the ACH levels at neutral pH. A negative correlation between ACH levels and *ALD5* expression was observed in RPMI biofilms incubated with D-glucose ($r_s=-0.763$, $p<0.001$). Similar negative correlations were observed in biofilms exposed to all conditions tested for ACH production from ethanol ($-0.769<r_s<-0.661$, $0.001<p<0.003$).

In contrast to neutral pH, most of the correlations between gene expression and ACH levels were not significant at acidic pH. In RPMI biofilms the *ALD5* expression correlated positively with ACH values after ethanol incubation ($r_s=0.746$, $p<0.001$) while expression of both *ADH1* and *ADH2* correlated negatively with ACH values ($r_s=-0.558$, $p=0.016$ and $r_s=-0.636$, $p=0.005$ respectively). This suggests a strong induction of pyruvate bypass towards acetate in RPMI biofilms at acidic pH as expected and control of pyruvate bypass by downstream metabolism genes, such as *ALD5*.

HICA treatment caused structural defects to intact *C. albicans* biofilms

Major defects were observed in biofilm ultrastructure after exposure to HICA particularly at acidic pH. Hyphal networks had collapsed and hyphae appeared fractured (Fig. 5A). A similarly sparse hyphal network was observed at neutral pH (Fig. 5B). In control images, hyphae appeared intact and unaffected by acidic pH (Fig. 5C, D). On exposure to HICA, more yeast cells were observed at neutral pH while hyphal tips showed signs of damage at acidic pH. Interestingly, when the HICA concentration was increased, more damaged hyphae were observed at acidic pH and the yeast cell phenotype was pronounced at neutral pH (figures not shown).

Discussion

Our study demonstrates a robust efficacy of HICA against *C. albicans* biofilms *in vitro*. Biofilm metabolic activity and biomass were significantly reduced by HICA especially under acidic conditions. The effect was visualized by SEM, which showed collapsing and aberrant hyphal structures in the pre-formed biofilms after exposure to HICA at both acidic and neutral pH conditions. There were signs of cell lysis in acidic conditions. A similar effect has been noted in *Aspergillus fumigatus* microcolonies in response to echinocandins [65]. In the present study there was no reduction of biomass when *C. albicans* biofilms were exposed to caspofungin, and the metabolic activity was reduced in early (4 h) biofilms only. In older biofilms, caspofungin exposure moderately increased the biofilm biomass thus supporting findings on the paradoxical effect on growth [41]. The effect of leucine on biomass, metabolic activity and ACH metabolism was opposite to that of HICA, and non-inhibitory at neutral pH, suggesting a different metabolic action. In contrast, cysteine exposure reduced the biofilm biomass as effectively as HICA at acidic pH, although no reduction in metabolic activity was observed. Cysteine is vital for cellular function, but high levels cause accumulation of toxic sulfite [66]. This is known to be toxic to bacteria and fungi but its impact on biofilms has not been studied [67–69]. Although a very low concentration of ethanol (0.05%) was used in this study a reduction in biofilm biomass of 33% at pH 5.2 and 22% at pH 7.4 was observed. This is of clinical interest as ethanol is used in antifungal lock therapy against *Candida* spp. biofilms [70].

In the human body ACH is metabolized into acetate mainly by the mitochondrial Ald enzyme (ALDH2; [71]). In our study, *ALD5* was the only Ald enzyme encoding gene highly expressed in *C. albicans* biofilms in all conditions tested. *C. albicans ALD5* is an ortholog of *S. cerevisiae ALD5*, which encodes the mitochondrial Ald enzyme [20,72]. Previously our group showed that *ALD6*, encoding the cytosolic counterpart, is highly expressed in hypoxic conditions in planktonically grown cells and the expression correlated well with ACH levels [25]. In early biofilms exposed to cysteine and PBS, a mild increase in the basal expression of *ALD6* relative to the reference gene was observed thus supporting the finding of planktonic cultures (data not shown). There was a correlation between ACH production and *ALD5* expression in our study. Impairment of pyruvate bypass downstream metabolism by down-regulation of *ALD5* was observed together with high ACH levels in caspofungin biofilms. *ADH1* was also highly expressed but

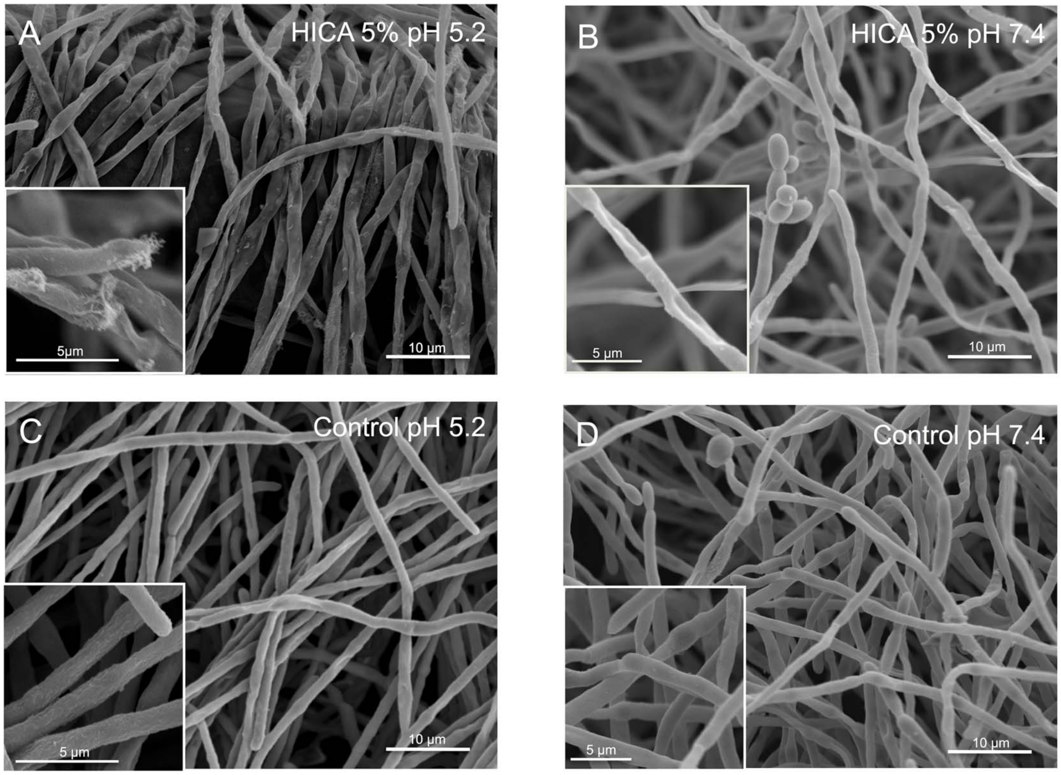

Figure 5. Microscopic examination of *C. albicans* biofilms exposed to HICA. SEM images were taken of biofilms which were grown for 24 h in RPMI at pH 7.4 and then exposed to 5% HICA for another 24 h at pH 5.2 (A) or pH 7.4 (B). Control images were taken of biofilms grown in RPMI-medium without HICA at pH 5.2 (C) or pH 7.4 (D). Insets show hyphal structures in detail. Scale bars indicate 10 μm in main images and 5 μm in insets.
doi:10.1371/journal.pone.0097864.g005

not in all conditions. On exposure to caspofungin, both *ADH1* and *ADH2* were down-regulated potentially resulting in ACH accumulation. This down-regulation correlates with the results of a previous study on 30 h *C. albicans* biofilms [73]. The positive correlation between expression of *ADH1* and *ADH2* seen in this study implies a functional role of Adh2p alongside Adh1p. This finding is in line with previous work on planktonic cultures in hypoxia [25]. The highest levels of *ADH1* expression were observed after exposure to PBS, HICA and cysteine and mainly at acidic pH. Gene expression from PBS biofilms reflected an induction of fermentation and pyruvate bypass as many genes in the pathway were highly expressed. Considering the toxicity of HICA and cysteine particularly at acidic pH, the up-regulation of *ADH1* and *ALD5* could be a response to oxidative stress and impairment of other respiratory functions. Interestingly, up-regulation of *ADH1* has been noted in apoptosis [74]. The significantly high basal expression of *ADH1* and *ALD5* in this study highlights their role in biofilms and supports the findings by others *in vitro* and *in vivo* [30,31,36].

Alcohol abuse is the main etiologic agent in upper digestive tract carcinogenesis and multiple studies by our group and others have shown that the carcinogenic effect of alcohol is a result of microbial metabolism of ethanol to ACH *in vitro* and *in vivo* [18,75–77]. Also, marked production of ACH by *Candida spp.* from D-glucose has been shown *in vitro* [15,18]. Considering that infections are mostly biofilm-related and to support our hypothesis of the carcinogenicity of *Candida* infection, it was important to elucidate the mutagenic potential of *C. albicans* biofilms and the expression of genes related to ACH and ethanol. Mutagenic levels of ACH were produced by control biofilms during incubation in D-glucose at neutral pH (up to 54 μM). No ACH was produced by biofilms exposed to HICA during D-glucose incubation at acidic pH. At neutral pH, the ACH levels were significantly lower compared to control (RPMI) conditions and below the mutagenic level (< 40 μM). At neutral pH, ACH production from ethanol or D-glucose by mature biofilms was decreased similarly by both HICA and caspofungin. Interestingly, although caspofungin inhibited ACH production by early biofilms more than HICA, it resulted in the highest ACH levels at acidic pH during D-glucose incubation. This is relevant as a normal western diet is often rich in D-glucose and the 100 mM concentration used in this study is equivalent to 18 g/l found commonly in food and beverages. In the presence of ethanol, ACH levels produced by all biofilms exposed to HICA or caspofungin were generally lower than in control biofilms. The ethanol concentration of 11 mM used in this study is found in saliva after drinking 0.5 g of alcohol per kg body weight, which is equivalent to 3 glasses of wine for an 80 kg male and thus can be considered clinically relevant. Our results on biofilms support earlier studies on planktonic cell cultures [12,15,18,77]. Biofilms exposed to RPMI and ethanol produced high levels of ACH from ethanol (up to 135 μM), well above the mutagenic level, and approaching the results obtained with planktonic cultures. It is relevant to point out that the highest ACH levels were produced at later stages of biofilm growth, as would be established in niches of the human body.

HICA was most active against *C. albicans* biofilms at acidic pH, in line with previous studies where planktonic cultures were used [43,44]. The main focus of the present study was to investigate the anti-biofilm efficacy of HICA against various comparators whereby it was critical to grow the biofilms under standardised conditions before treatment at acidic and neutral conditions. Therefore, the biofilms were not pre-grown at acidic conditions, which can be considered a limitation: there are multiple sites that are physiologically acidic and pH can have an effect on C. albicans morphology [78]. On the other hand, acidic pH may favour fungal over bacterial growth as seen in *Candida* esophagitis, vulvovaginal candidosis and chronic wounds [79–83]. Interestingly, the final metabolic activity and biomass of untreated (RPMI) control biofilms after 24 h exposure to pH 5.2 and 7.4 were comparable in the present study.

A recent report suggested that composition of the medium has a major effect on biofilm architecture, expression profiles and antifungal susceptibility [84]. In RPMI medium, the D-glucose concentration of 2.0 g/l is higher than the physiological concentration normally found in the human body (0.7–1.0 g/l; [85]). Thus adjustment of the D-glucose concentration could bring the *in vitro* models closer to physiological conditions in the human body and allow for the discovery of the effects that may be masked by the artificially high D-glucose concentrations used *in vitro*. Also a major disadvantage of our study and multiple others is the use of only one strain. There are major differences between clinical and reference strains with respect to metabolic activity, susceptibility profiles and the ability to form biofilms which suggests this analysis would benefit by confirmation with clinical isolates [86]. It is important to note that ACH levels were not standardised to cell count or biomass but per biofilm unit. This was in order to avoid potential artifacts that would arise through biofilm killing by antifungal agents.

The mode of action for HICA is still unknown, and broader transcriptional and proteomic studies are warranted to elucidate the specific factors underlying its anti-microbial activity. Considering the similar expression of ACH catabolism genes after cysteine and HICA exposure, cysteine may provide clues in the quest to understand the mode of action of HICA. A few *Lactobacillus*-fungus co-culture studies with a transcriptomic approach have already shed light on this inhibitory mechanism and they indicate a global metabolic shutdown in fungal cells in response to *Lactobacillus* metabolites [50,52]. Considering the urgent need for effective treatment strategies against fungal infections, HICA could provide an alternative and effective approach in the fight against superficial *Candida* biofilm infections in concentrations relevant to topical treatment or lock therapies. Together with its broadly antibacterial activity it could also be a good choice for mixed bacterial-fungal infections. The decreased mutagenic potential observed in biofilms exposed to HICA would suggest that long-term therapy would not cause harmful exposure of adjacent mucosa and surrounding bacteria to mutagenic ACH although more studies are required to confirm this.

Supporting Information

Table S1 List of primers used in this study.
(DOCX)

Acknowledgments

We thank Keely Young for technical assistance and Dr. Julie Morris for her expert guidance on statistical analysis.

Author Contributions

Conceived and designed the experiments: MN LNF GR PB R. Rautemaa. Performed the experiments: MN LNF VR R. Rajendran. Analyzed the data: MN LNF VR GR PB R. Rautemaa. Contributed reagents/materials/analysis tools: TS GR PB R. Rautemaa. Wrote the paper: MN LNF R. Rajendran TS GR PB R. Rautemaa.

References

1. Brown GD, Denning DW, Gow NA, Levitz SM, Netea MG, et al. (2012) Hidden killers: human fungal infections. Sci Transl Med 4: 165rv113.
2. Costerton JW, Stewart PS, Greenberg EP (1999) Bacterial biofilms: a common cause of persistent infections. Science 284: 1318–1322.
3. Ramage G, Mowat E, Jones B, Williams C, Lopez-Ribot J (2009) Our current understanding of fungal biofilms. Crit Rev Microbiol 35: 340–355.
4. Taff HT, Mitchell KF, Edward JA, Andes DR (2013) Mechanisms of *Candida* biofilm drug resistance. Future Microbiol 8: 1325–1337.
5. Baillie GS, Douglas LJ (1999) *Candida* biofilms and their susceptibility to antifungal agents. Methods Enzymol 310: 644–656.
6. Ramage G, VandeWalle K, Lopez-Ribot JL, Wickes BL (2002) The filamentation pathway controlled by the Efg1 regulator protein is required for normal biofilm formation and development in *Candida albicans*. FEMS Microbiol Lett 214: 95–100.
7. Land GA, McDonald WC, Stjernholm RL, Friedman L (1975) Factors affecting filamentation in *Candida albicans*: changes in respiratory activity of *Candida albicans* during filamentation. Infect Immun 12: 119–127.
8. Setiadi ER, Doedt T, Cottier F, Noffz C, Ernst JF (2006) Transcriptional response of *Candida albicans* to hypoxia: linkage of oxygen sensing and Efg1p-regulatory networks. J Mol Biol 361: 399–411.
9. Vellucci VF, Gygax SE, Hostetter MK (2007) Involvement of *Candida albicans* pyruvate dehydrogenase complex protein X (Pdx1) in filamentation. Fungal Genet Biol 44: 979–990.
10. Vylkova S, Carman AJ, Danhof HA, Collette JR, Zhou H, et al. (2011) The fungal pathogen *Candida albicans* autoinduces hyphal morphogenesis by raising extracellular pH. MBio 2: e00055–00011.
11. Coussens LM, Werb Z (2002) Inflammation and cancer. Nature 420: 860–867.
12. Marttila E, Uittamo J, Rusanen P, Lindqvist C, Salaspuro M, et al. (2013) Acetaldehyde production and microbial colonization in oral squamous cell carcinoma and oral lichenoid disease. Oral Surg Oral Med Oral Pathol Oral Radiol 116: 61–68.
13. Rautemaa R, Hietanen J, Niissalo S, Pirinen S, Perheentupa J (2007) Oral and oesophageal squamous cell carcinoma—a complication or component of autoimmune polyendocrinopathy-candidiasis-ectodermal dystrophy (APECED, APS-I). Oral Oncol 43: 607–613.
14. Rautemaa R, Ramage G (2011) Oral candidosis—clinical challenges of a biofilm disease. Crit Rev Microbiol 37: 328–336.
15. Uittamo J, Siikala E, Kaihovaara P, Salaspuro M, Rautemaa R (2009) Chronic candidosis and oral cancer in APECED-patients: production of carcinogenic acetaldehyde from glucose and ethanol by *Candida albicans*. Int J Cancer 124: 754–756.
16. Secretan B, Straif K, Baan R, Grosse Y, El Ghissassi F, et al. (2009) A review of human carcinogens—Part E: tobacco, areca nut, alcohol, coal smoke, and salted fish. Lancet Oncol 10: 1033–1034.
17. Brooks PJ, Theruvathu JA (2005) DNA adducts from acetaldehyde: implications for alcohol-related carcinogenesis. Alcohol 35: 187–193.
18. Nieminen MT, Uittamo J, Salaspuro M, Rautemaa R (2009) Acetaldehyde production from ethanol and glucose by non-*Candida albicans* yeasts *in vitro*. Oral Oncol 45: e245–248.
19. Flores CL, Rodriguez C, Petit T, Gancedo C (2000) Carbohydrate and energy-yielding metabolism in non-conventional yeasts. FEMS Microbiol Rev 24: 507–529.
20. Inglis DO, Arnaud MB, Binkley J, Shah P, Skrzypek MS, et al. (2012) The *Candida* genome database incorporates multiple *Candida* species: multispecies search and analysis tools with curated gene and protein information for *Candida albicans* and *Candida glabrata*. Nucleic Acids Res 40: D667–674.
21. Bertram G, Swoboda RK, Gooday GW, Gow NA, Brown AJ (1996) Structure and regulation of the *Candida albicans ADH1* gene encoding an immunogenic alcohol dehydrogenase. Yeast 12: 115–127.
22. Mukherjee PK, Mohamed S, Chandra J, Kuhn D, Liu S, et al. (2006) Alcohol dehydrogenase restricts the ability of the pathogen *Candida albicans* to form a biofilm on catheter surfaces through an ethanol-based mechanism. Infect Immun 74: 3804–3816.
23. Thomas DP, Viudes A, Monteagudo C, Lazzell AL, Saville SP, et al. (2006) A proteomic-based approach for the identification of *Candida albicans* protein components present in a subunit vaccine that protects against disseminated candidiasis. Proteomics 6: 6033–6041.
24. Maestre O, Garcia-Martinez T, Peinado RA, Mauricio JC (2008) Effects of ADH2 overexpression in *Saccharomyces bayanus* during alcoholic fermentation. Appl Environ Microbiol 74: 702–707.
25. Marttila E, Bowyer P, Sanglard D, Uittamo J, Kaihovaara P, et al. (2013) Fermentative 2-carbon metabolism produces carcinogenic levels of acetaldehyde in *Candida albicans*. Mol Oral Microbiol 28: 281–291.
26. Nobile CJ, Nett JE, Hernday AD, Homann OR, Deneault JS, et al. (2009) Biofilm matrix regulation by *Candida albicans* Zap1. PLoS Biol 7: e1000133.
27. Cherry JM, Hong EL, Amundsen C, Balakrishnan R, Binkley G, et al. (2012) Saccharomyces Genome Database: the genomics resource of budding yeast. Nucleic Acids Res 40: D700–705.
28. Strijbis K, van Roermund CW, Hardy GP, van den Burg J, Bloem K, et al. (2009) Identification and characterization of a complete carnitine biosynthesis pathway in *Candida albicans*. FASEB J 23: 2349–2359.
29. Strijbis K, van Roermund CW, Visser WF, Mol EC, van den Burg J, et al. (2008) Carnitine-dependent transport of acetyl coenzyme A in *Candida albicans* is essential for growth on nonfermentable carbon sources and contributes to biofilm formation. Eukaryot Cell 7: 610–618.
30. Martinez-Gomariz M, Perumal P, Mekala S, Nombela C, Chaffin WL, et al. (2009) Proteomic analysis of cytoplasmic and surface proteins from yeast cells, hyphae, and biofilms of *Candida albicans*. Proteomics 9: 2230–2252.
31. Seneviratne CJ, Wang Y, Jin L, Abiko Y, Samaranayake LP (2008) *Candida albicans* biofilm formation is associated with increased anti-oxidative capacities. Proteomics 8: 2936–2947.
32. Stichternoth C, Ernst JF (2009) Hypoxic adaptation by Efg1 regulates biofilm formation by *Candida albicans*. Appl Environ Microbiol 75: 3663–3672.
33. Carman AJ, Vylkova S, Lorenz MC (2008) Role of acetyl coenzyme A synthesis and breakdown in alternative carbon source utilization in *Candida albicans*. Eukaryot Cell 7: 1733–1741.
34. Hernaez ML, Ximenez-Embun P, Martinez-Gomariz M, Gutierrez-Blazquez MD, Nombela C, et al. (2010) Identification of *Candida albicans* exposed surface proteins *in vivo* by a rapid proteomic approach. J Proteomics 73: 1404–1409.
35. Monteoliva L, Martinez-Lopez R, Pitarch A, Hernaez ML, Serna A, et al. (2011) Quantitative proteome and acidic subproteome profiling of *Candida albicans* yeast-to-hypha transition. J Proteome Res 10: 502–517.
36. Pierce JV, Dignard D, Whiteway M, Kumamoto CA (2013) Normal adaptation of *Candida albicans* to the murine gastrointestinal tract requires Efg1p-dependent regulation of metabolic and host defense genes. Eukaryot Cell 12: 37–49.
37. Cornely OA, Bassetti M, Calandra T, Garbino J, Kullberg BJ, et al. (2012) ESCMID* guideline for the diagnosis and management of *Candida* diseases 2012: non-neutropenic adult patients. Clin Microbiol Infect 18 Suppl 7: 19–37.
38. Deresinski SC, Stevens DA (2003) Caspofungin. Clin Infect Dis 36: 1445–1457.
39. Pfaller M, Boyken L, Hollis R, Kroeger J, Messer S, et al. (2011) Use of epidemiological cutoff values to examine 9-year trends in susceptibility of *Candida* species to anidulafungin, caspofungin, and micafungin. J Clin Microbiol 49: 624–629.
40. Pfaller MA, Boyken L, Hollis RJ, Messer SA, Tendolkar S, et al. (2006) *In vitro* susceptibilities of *Candida* spp. to caspofungin: four years of global surveillance. J Clin Microbiol 44: 760–763.
41. Stevens DA, Espiritu M, Parmar R (2004) Paradoxical effect of caspofungin: reduced activity against *Candida albicans* at high drug concentrations. Antimicrob Agents Chemother 48: 3407–3411.
42. Walker LA, Gow NA, Munro CA (2013) Elevated chitin content reduces the susceptibility of *Candida* species to caspofungin. Antimicrob Agents Chemother 57: 146–154.
43. Sakko M, Moore C, Novak-Frazer L, Rautemaa V, Sorsa T, et al. (2013) 2-hydroxyisocaproic acid is fungicidal for *Candida* and *Aspergillus* species. Mycoses 57:214–221.
44. Sakko M, Tjaderhane L, Sorsa T, Hietala P, Jarvinen A, et al. (2012) 2-Hydroxyisocaproic acid (HICA): a new potential topical antibacterial agent. Int J Antimicrob Agents 39: 539–540.
45. Guo J, Brosnan B, Furey A, Arendt E, Murphy P, et al. (2012) Antifungal activity of Lactobacillus against *Microsporum canis*, *Microsporum gypseum* and *Epidermophyton floccosum*. Bioeng Bugs 3: 104–113.
46. Hietala PK, Westermarck HW, Jaarma M (1979) Identification of antimicrobial alpha-hydroxyacids in *Lactobacillus plantarum*-fermented animal protein. Nutr Metab 23: 227–234.
47. Mero AA, Ojala T, Hulmi JJ, Puurtinen R, Karila TA, et al. (2010) Effects of alfa-hydroxy-isocaproic acid on body composition, DOMS and performance in athletes. J Int Soc Sports Nutr 7: 1.
48. Boebel KP, Baker DH (1982) Comparative utilization of the alpha-keto and D- and L-alpha-hydroxy analogs of leucine, isoleucine and valine by chicks and rats. J Nutr 112: 1929–1939.
49. Westermarck HW, Hietala P, Jaarma M, Sorsa T, Vaara M (1997) Use of alpha-hydroxy acids in the manufacture of a medicament for the treatment of inflammation. European Patent Office. Patent no.EP0871438B1.
50. Crowley S, Mahony J, van Sinderen D (2012) Comparative analysis of two antifungal *Lactobacillus plantarum* isolates and their application as bioprotectants in refrigerated foods. J Appl Microbiol 113: 1417–1427.
51. Crowley S, Mahony J, van Sinderen D (2013) Broad-spectrum antifungal-producing lactic acid bacteria and their application in fruit models. Folia Microbiol (Praha) 58: 291–299.
52. Kohler GA, Assefa S, Reid G (2012) Probiotic interference of *Lactobacillus rhamnosus* GR-1 and *Lactobacillus reuteri* RC-14 with the opportunistic fungal pathogen *Candida albicans*. Infect Dis Obstet Gynecol 2012: 636474.
53. Bachmann SP, VandeWalle K, Ramage G, Patterson TF, Wickes BL, et al. (2002) *In vitro* activity of caspofungin against *Candida albicans* biofilms. Antimicrob Agents Chemother 46: 3591–3596.
54. Ramage G, Milligan S, Lappin DF, Sherry L, Sweeney P, et al. (2012) Antifungal, cytotoxic, and immunomodulatory properties of tea tree oil and its derivative components: potential role in management of oral candidosis in cancer patients. Front Microbiol 3: 220.
55. Ahn SJ, Costa J, Emanuel JR (1996) PicoGreen quantitation of DNA: effective evaluation of samples pre- or post-PCR. Nucleic Acids Res 24: 2623–2625.

56. Marstorp H, Witter E (1999) Extractable dsDNA and product formation as measures of microbial growth in soil upon substrate addition. Soil Biol Biochem 31: 1443–1453.
57. Ramage G, Lopez-Ribot JL (2005) Techniques for antifungal susceptibility testing of *Candida albicans* biofilms. Methods Mol Med 118: 71–79.
58. Saravanan P, Pakshirajan K, Saha P (2009) Batch growth kinetics of an indigenous mixed microbial culture utilizing m-cresol as the sole carbon source. J Hazard Mater 162: 476–481.
59. Gillum AM, Tsay EY, Kirsch DR (1984) Isolation of the *Candida albicans* gene for orotidine-5′-phosphate decarboxylase by complementation of *S. cerevisiae ura3* and *E. coli pyrF* mutations. Mol Gen Genet 198: 179–182.
60. Wilson K (1987) Large scale CsCl preparation of genomic DNA from bacteria. In: Ausubel FMR, Brent, R.E.,Kingston, D.D., Moore, J.G., Seidman, J.A., Smith, K. . Struhl, editor. Current Protocols in Molecular Biology. New York: Greene Publishing Associates and Wiley Interscience. 2.4.3–2.4.5.
61. Untergasser A, Nijveen H, Rao X, Bisseling T, Geurts R, et al. (2007) Primer3Plus, an enhanced web interface to Primer3. Nucleic Acids Res 35: W71–74.
62. Nailis H, Coenye T, Van Nieuwerburgh F, Deforce D, Nelis HJ (2006) Development and evaluation of different normalization strategies for gene expression studies in *Candida albicans* biofilms by real-time PCR. BMC Mol Biol 7: 25.
63. Pfaffl MW, Horgan GW, Dempfle L (2002) Relative expression software tool (REST) for group-wise comparison and statistical analysis of relative expression results in real-time PCR. Nucleic Acids Res 30: e36.
64. Erlandsen SL, Kristich CJ, Dunny GM, Wells CL (2004) High-resolution visualization of the microbial glycocalyx with low-voltage scanning electron microscopy: dependence on cationic dyes. J Histochem Cytochem 52: 1427–1435.
65. Ingham CJ, Schneeberger PM (2012) Microcolony imaging of *Aspergillus fumigatus* treated with echinocandins reveals both fungistatic and fungicidal activities. PLoS One 7: e35478.
66. Hennicke F, Grumbt M, Lermann U, Ueberschaar N, Palige K, et al. (2013) Factors supporting cysteine tolerance and sulfite production in *Candida albicans*. Eukaryot Cell 12: 604–613.
67. Park S, Imlay JA (2003) High levels of intracellular cysteine promote oxidative DNA damage by driving the fenton reaction. J Bacteriol 185: 1942–1950.
68. Wain WH, Price MF, Cawson RA (1975) A re-evaluation of the effect of cysteine or *Candida albicans*. Sabouraudia 13 Pt 1: 74–82.
69. Winsten S, Murray TJ (1956) Virulence enhancement of a filamentous strain of *Candida albicans* after growth on media containing cysteine. J Bacteriol 71: 738.
70. Walraven CJ, Lee SA (2013) Antifungal lock therapy. Antimicrob Agents Chemother 57: 1–8.
71. Peng GS, Yin SJ (2009) Effect of the allelic variants of aldehyde dehydrogenase *ALDH2*2* and alcohol dehydrogenase *ADH1B*2* on blood acetaldehyde concentrations. Hum Genomics 3: 121–127.
72. Saint-Prix F, Bonquist L, Dequin S (2004) Functional analysis of the *ALD* gene family of *Saccharomyces cerevisiae* during anaerobic growth on glucose: the NADP+-dependent Ald6p and Ald5p isoforms play a major role in acetate formation. Microbiology 150: 2209–2220.
73. Vediyappan G, Rossignol T, d'Enfert C (2010) Interaction of *Candida albicans* biofilms with antifungals: transcriptional response and binding of antifungals to beta-glucans. Antimicrob Agents Chemother 54: 2096–2111.
74. Shirtliff ME, Krom BP, Meijering RA, Peters BM, Zhu J, et al. (2009) Farnesol-induced apoptosis in *Candida albicans*. Antimicrob Agents Chemother 53: 2392–2401.
75. Homann N, Jousimies-Somer H, Jokelainen K, Heine R, Salaspuro M (1997) High acetaldehyde levels in saliva after ethanol consumption: methodological aspects and pathogenetic implications. Carcinogenesis 18: 1739–1743.
76. Salaspuro M (2009) Acetaldehyde as a common denominator and cumulative carcinogen in digestive tract cancers. Scand J Gastroenterol 44: 912–925.
77. Uittamo J, Nieminen MT, Kaihovaara P, Bowyer P, Salaspuro M, et al. (2010) Xylitol inhibits carcinogenic acetaldehyde production by *Candida* species. Int J Cancer 129: 2038–2041.
78. Kim J, Sudbery P (2011) *Candida albicans*, a major human fungal pathogen. J Microbiol 49: 171–177.
79. Dowd SE, Delton Hanson J, Rees E, Wolcott RD, Zischau AM, et al. (2011) Survey of fungi and yeast in polymicrobial infections in chronic wounds. J Wound Care 20: 40–47.
80. Mousa HA, Al-Bader SM, Hassan DA (1999) Correlation between fungi isolated from burn wounds and burn care units. Burns 25: 145–147.
81. Odds FC (1979) *Candida* and candidosis Baltimore: University Park Press. 382 p.
82. Samaranayake LP, Keung Leung W, Jin L (2009) Oral mucosal fungal infections. Periodontol 2000 49: 39–59.
83. Sobel JD (2007) Vulvovaginal candidosis. Lancet 369: 1961–1971.
84. Kucharikova S, Tournu H, Lagrou K, Van Dijck P, Bujdakova H (2011) Detailed comparison of *Candida albicans* and *Candida glabrata* biofilms under different conditions and their susceptibility to caspofungin and anidulafungin. J Med Microbiol 60: 1261–1269.
85. Tirosh A, Shai I, Tekes-Manova D, Israeli E, Pereg D, et al. (2005) Normal fasting plasma glucose levels and type 2 diabetes in young men. N Engl J Med 353: 1454–1462.
86. Tumbarello M, Fiori B, Trecarichi EM, Posteraro P, Losito AR, et al. (2012) Risk factors and outcomes of candidemia caused by biofilm-forming isolates in a tertiary care hospital. PLoS One 7: e33705.
